# Immunolocalization of pectic polysaccharides during abscission in pea seeds (*Pisum sativum* L.) and in abscission less *def* pea mutant seeds

**DOI:** 10.1186/s13104-016-2231-z

**Published:** 2016-08-31

**Authors:** YeonKyeong Lee, Kwadwo Owusu Ayeh, Mike Ambrose, Anne Kathrine Hvoslef-Eide

**Affiliations:** 1 Department of Plant Sciences, Norwegian University of Life Sciences (NMBU), P.O. BOX 5003, 1432 Ås, Norway; 2 Department of Botany, School of Biological Sciences, College of Basic and Applied Sciences, University of Ghana, Legon-Accra, Ghana; 3 Department of Crops Genetics, John Innes Centre, Norwich Research Park, Colney Lane, NR4 7UH Norwich, UK

**Keywords:** Pea (*Pisum sativum* L.), Abscission, *Def* mutant, Immunolocalization, Pectic polysaccharide

## Abstract

**Background:**

In pea seeds (*Pisum sativum* L.), the presence of the *Def* locus determines abscission event between its funicle and the seed coat. Cell wall remodeling is a necessary condition for abscission of pea seed. The changes in cell wall components in wild type (WT) pea seed with *Def* loci showing seed abscission and in abscission less *def* mutant peas were studied to identify the factors determining abscission and non-abscission event.

**Methods:**

Changes in pectic polysaccharides components were investigated in WT and *def* mutant pea seeds using immunolabeling techniques. Pectic monoclonal antibodies (1 → 4)-β-d-galactan (LM5), (1 → 5)-α-l-arabinan(LM6), partially de-methyl esterified homogalacturonan (HG) (JIM5) and methyl esterified HG (JIM7) were used for this study.

**Results:**

Prior to abscission zone (AZ) development, galactan and arabinan reduced in the predestined AZ of the pea seed and disappeared during the abscission process. The AZ cells had partially de-methyl esterified HG while other areas had highly methyl esterified HG. A strong JIM5 labeling in the *def* mutant may be related to cell wall rigidity in the mature *def* mutants. In addition, the appearance of pectic epitopes in two F_3_ populations resulting from cross between WT and *def* mutant parents was studied. As a result, we identified that homozygous dominant lines (*Def*/*Def*) showing abscission and homozygous recessive lines (*def*/*def*) showing non-abscission had similar immunolabeling pattern to their parents. However, the heterogeneous lines (*Def/def*) showed various immunolabeling pattern and the segregation pattern of the *Def* locus.

**Conclusions:**

Through the study of the complexity and variability of pectins in plant cell walls as well as understanding the segregation patterns of the *Def* locus using immunolabeling techniques, we conclude that cell wall remodeling occurs in the abscission process and de-methyl esterification may play a role in the non-abscission event in *def* mutant. Overall, this study contributes new insights into understanding the structural and architectural organization of the cell walls during abscission.

## Background

Abscission is an intriguing process to study that involves the shedding of plant organs such as leaves, petals, sepals, stamens, styles, fruits and seeds [1, 2]. The abscission process may be formed by active cell division to form an abscission layer as in flower pedicel in poinsettia [3] or there could be a preformed separation layer leading the process of abscission as seen in pea seeds [4]. Roberts et al. [5] revealed that the disassembly of cell wall components formed the primary events in the separation layer during the abscission process. A wealth of valuable information regarding cell wall-modifying proteins such as endoglucanases [6, 7], expansin [8, 9] and polygalacturonases [10–12] during the abscission process has been reported. Although the abscission event is a natural process, early abscission of pea seeds may result in yield loss. Here we adopt *def* mutant pea that does not show seed abscission from the funicle to identify the mechanical process especially relating to cell wall modification in the abscission process.

In *Def* WT pea (*Pisum sativum* L.), the abscission of the seed from the funicle takes place beneath a counter palisade layer (CPL) delimiting the embryo and the funicle. In many of the *Fabaceae* family members, the epidermal layer of most seeds of legume takes their origin from the outermost integument and is composed of a single layer of macrosclereid palisade cells [13]. A palisade layer characterises the epidermal layer within the seed coat. However, towards the hilum region, the CPL emerges [14], originating from the funiculus and this CPL is fused with the palisade layer, forming double layers at the hilum in the WT pea seeds [15, 16]. In contrast, a spontaneous *def* mutant [17] has the seed strongly attached to the funicle and the subtending seed does not abscise from the funicle at any point in time. The double palisade layers have been suggested to play a structural supporting role in the attachment of the seed to the funicle [18].

Pectins are a group of complex polysaccharides with the distinctive characteristic of being heterogenous, branched and highly hydrated polysaccharides. Pectins are major components in plant cell walls and exclusively located in the primary cell walls [19–21]. Pectins have played key roles in the cell wall mechanical properties [22–24], cell differentiation and cell growth [25, 26]. Pectins are composed of homogalacturonan (HG), xylogalacturonan (XGA), rhamnogalacturonan-I (RG-I), and rhamnogalacturonan-II (RG-II) and the polysaccharides consists mostly of neutral sugars, such as arabinan, galactan, and arabinogalactan [27–30]. HG is a linear homopolymer of (1 → 4)-linked-α-d-galacturonic acid (Gal A) and it is the most abundant pectic polymer. Naturally, HG is synthesized with methyl group. The GalA residues can be partially methyl-esterified [28] or acetylated [31] and in some cases both [32]. De-methyl esterified or partially methyl esterified HGs readily form calcium cross-linked gels [33] and results in a stiffer material and altering the mechanical properties of the cell walls [30]. Thus, the degree of de-esterification of the HG may be a key positive regulator for cell adhesion or cell separation. The RG-I contains as many as 100 repeats and consists of (1 → 2)-linked-α-l-rhamnose-(1 → 4)-linked-α-d-galacturonic acid [34]. RG-I often have side chains of polysaccharides notably galactan, arabinan and arabinogalactan attached to their C4 position (1 → 2)-linked-α-l-rhamnose-(1 → 4)-linked-α-d-galacturonic acid [21, 35]. RG-II is a highly substituted and branched homogalacturonan, carrying complex and distinct side chains attached to the GalA residues [28, 36].

Monoclonal antibodies (mAbs) are extensively used for the analysis of pectins. They ensure that defined structural domains are localised and hence reveal intact cell wall architecture [23, 37, 38]. Thus, the monoclonal antibodies represent an array of powerful tools on pectic polysaccharides to complete biochemical knowledge and to understand the occurrence and function of the pectic polysaccharides [30]. In view of the immense complex nature of pectic polysaccharides, several anti-pectin monoclonal antibodies have been generated and characterized [39, 40].

In this study, we investigated the differential localization of the pectic epitopes in the WT pea with an abscission event and the *def* mutant with non-abscission. The specific purpose of this work was to map the distribution of pectic epitopes in the WT pea seeds during abscission process and non-abscission in the *def* mutant seeds. Furthermore, we studied the distribution of pectic epitopes in two F_3_ populations obtained from cross between WT (*Def*) and mutant (*def*) parents since it showed variation in their abscission event [41]. We aim to define the physical mechanisms underlying cell adhesion and cell separation in the pea seeds.

## Methods

### Plant materials

The four lines of pea (*Pisum sativum* L.) seeds (JI 116, JI 2822, JI 1184 and JI 3020) in this study were selected based on the presence of specific alleles at the *Def* locus, which control the detachment of the seed from the funicle [4]. Two WTs and two *def* mutant pea seeds were kindly supplied from the John Innes Pisum Collection (Table 1). Tall WT (JI 116) and dwarf WT (JI 2822) have a normal abscission of seeds. Tall *def* mutant (JI 1184) and dwarf *def* mutant (JI 3020) both lack the seed abscission. The seeds of each line were sown in pots with fertilised peat and grown under greenhouse conditions at 22 °C and 16/8 h photoperiod with a photon flux of 110 μmol m^−2^ s^−1^ (400–700 nm photosynthetic active radiation) and a day length extending light provided from incandescent lamps (OSRAM, Germany).

**Table 1:** Details of *Pisum sativum* L. accessions and their allelic status with respect to the *Def* locus

Accession	Name	*Def* allele	Phenotype
JI 116	cv. Parvus	*Def* (wild type)	Tall
JI 2822	RIL, research line	*Def* (wild type)	Dwarf
JI 1184	Priekuskij-341-*def*	*def* (mutant)	Tall
JI 3020	cv. Nord	*def* (mutant)	Dwarf

### Definition of developmental stages

We used young and mature developmental stages for both the WT and the *def* mutant pea plants. For the tall WT JI 116, the developmental stage 10.1 indicates young seed. For the tall *def* mutant type JI 1184, the developmental stage 8.1 indicates young seed for a comparable developmental stage. The developmental stage 2.1 indicates mature seed for both WT JI 116 and mutant JI 1184. For the dwarf WT JI 2822 and the dwarf *def* mutant JI 3020, the developmental stages 4.1 and 3.1 indicate young seeds, respectively. The developmental stages 1.1 and 1.2 indicate mature seed for both the dwarf WT JI 2822 and the dwarf *def* mutant JI 3020, respectively. In F_3_ populations, the developmental stages 3.1 and 1.1 indicate young and mature seeds, respectively. The developmental stage 2.1 means an intermediary stage.

### Plant tissue preparation

For immunological analysis, seeds were embedded in LR White resin (London Resin Company, England) as previously mentioned [3]. The funicle-seed coat interface in seeds were cut into 2 mm-thickness. The cut materials were immediately fixed and vacuum infiltrated. Fixed and infiltrated tissues were placed at 4 °C overnight. The fixed samples were washed with phosphate-buffered saline (PBS) and then dehydrated in a graded ethanol series. Infiltration was performed with a progressively increasing ratio of the LR White resin to ethanol and the specimens were embedded in the LR White resin at the end of the infiltration process. The embedded plant materials were sectioned into 1 μm-thick sections. The sections were placed on Vectabond (Vector Laboratories, USA) coated glass slides and heated at 55 °C on a warm plate overnight to firmly adhere the sections to the slide.

### F_3_ plant materials

Segregation patterns in inheritance of the *Def* locus were studied in two F_3_ populations as previously described [41]. Selected seed lines from two F_3_ populations were produced from crosses between parents JI2822 (dwarf WT) × JI 1184 (tall *def* mutant) (population one) and JI 2822 (dwarf WT) × JI 3020 (dwarf *def* mutant) (population two). The F_1_ from the two populations were selfed to produce F_2_ plants. The F_2_ populations were grown under same conditions as described above to produce the F_3_ seeds in two populations, and then used for study.

### Histological characterization and immunoanalyses of cell wall polysaccharides

The sections were stained with toluidine blue O (Sigma, USA) for histological analysis. The stained materials were washed with distilled water and mounted in Depex (BDH, USA). The sections were examined using a Leica Brightfield microscope (Leica, Germany). The monoclonal antibodies used in this study are in Table 2 and detail methods for immunolabeling are previously explained in Lee et al. [3]. For indirect immunofluorescence labeling, the sections were incubated in milk protein/PBS to block non-specific binding. The sections were then incubated with diluted primary rat monoclonal antibody (1:10) for 1 h at room temperature. The sections were washed with PBS and incubated with the secondary antibody, anti-rat-IgG whole molecule linked to fluorescein isothiocyanate (Sigma, USA). The sections were examined with Leica microscope equipped with epifluorescence (Leica, Germany).

**Table 2:** Pectic polysaccharides antibodies used in this study

Mabs	Antigen/epitope	References
LM5	(1 → 4)-β-d-galactan	Jones et al. [39]
LM6	(1 → 5)-α-l-arabinan	Willats et al. [40]
JIM5	Partially methyl-esterified HG epitope; unesterified residues	Clausen et al. [56]
JIM7	Partially methyl-esterified HG epitope; methyl esterified residues	Clausen et al. [56]

## Results

**Fig. 1:**
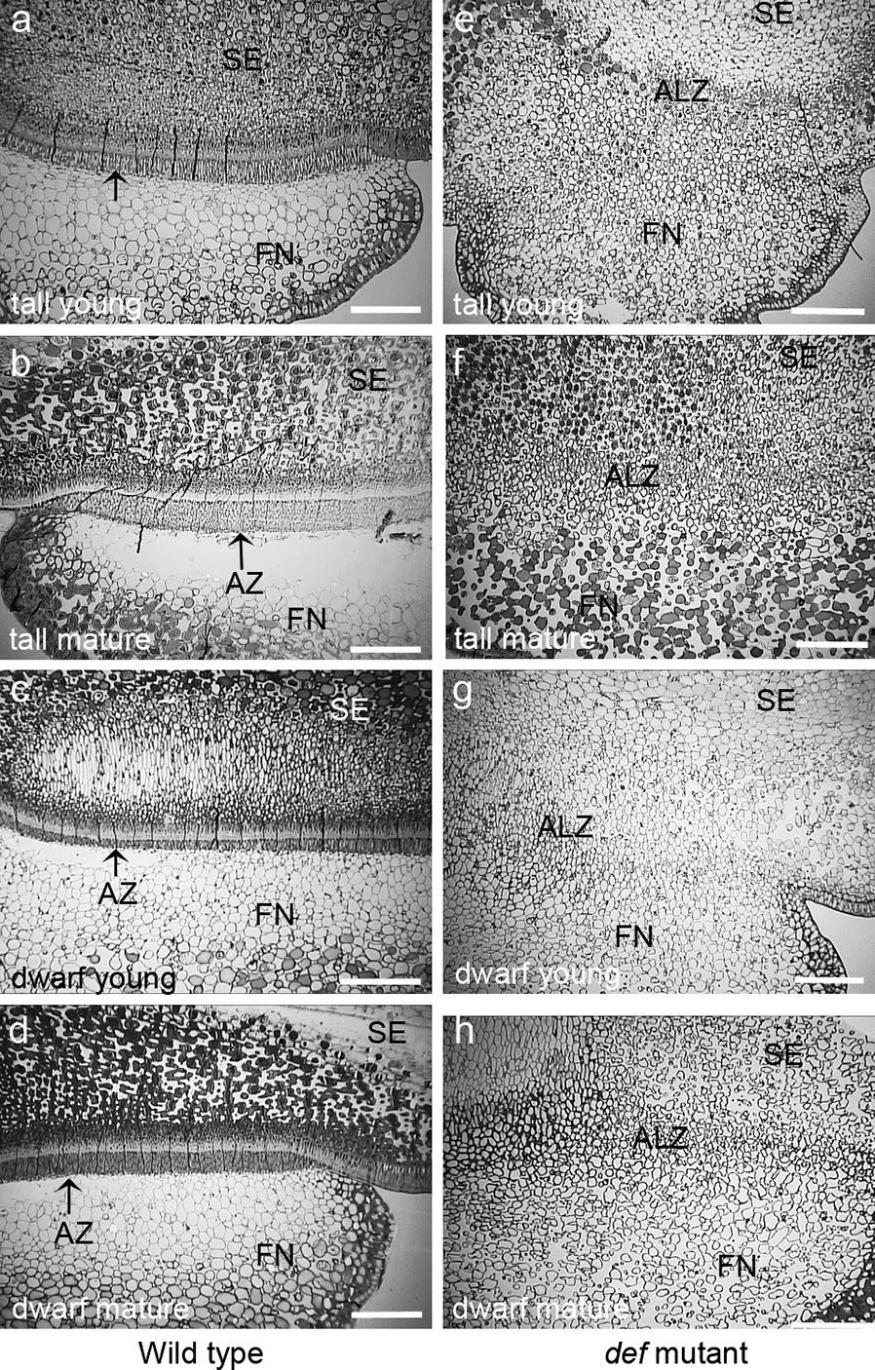
Light micrographs of pea seed sections stained with *toluidine blue* to show structural differences at intervening area between funicle and seed coat in wild type and *def* mutant peas. **a** JI116 tall WT young pea at 10.1. **b** JI116 tall WT mature pea at 2.1. **c** JI2822 dwarf WT young pea at 4.1. **d** JI2822 dwarf WT mature pea at 1.1. **e** JI1184 tall *def* mutant young pea at 8.1. **f** JI1184 tall *def* mutant mature pea at 2.1. **g** JI3020 dwart *def* mutant young pea at 3.1. **h** JI3020 dwarf *def* mutant mature pea at 1.2. *AZ* abscission zone; *FN* funicle; *SE* seed; *ALZ* abscission less zone. *Arrows* indicate the AZ. *Scale bars* 310 µm

We observed a clear abscission event in the WT pea seeds (Fig. 1a–d). The abscission was associated with a distinct double palisade layers at the interface between the seed coat and the funicle and the development of the AZ was dependent on seed maturity. The mature seeds showed fully developed AZ (Fig. 1b and d) which was not present in the young WT seeds (Fig. 1a). Figure 1c showed distinct AZ, although it was young stage of JI2822. However, the *def* mutant seeds did not show any abscission event at any developmental stage (Fig. 1e–h).

### Pectic polysaccharide distribution during the abscission process in WT and *def* mutant pea

**Fig. 2:**
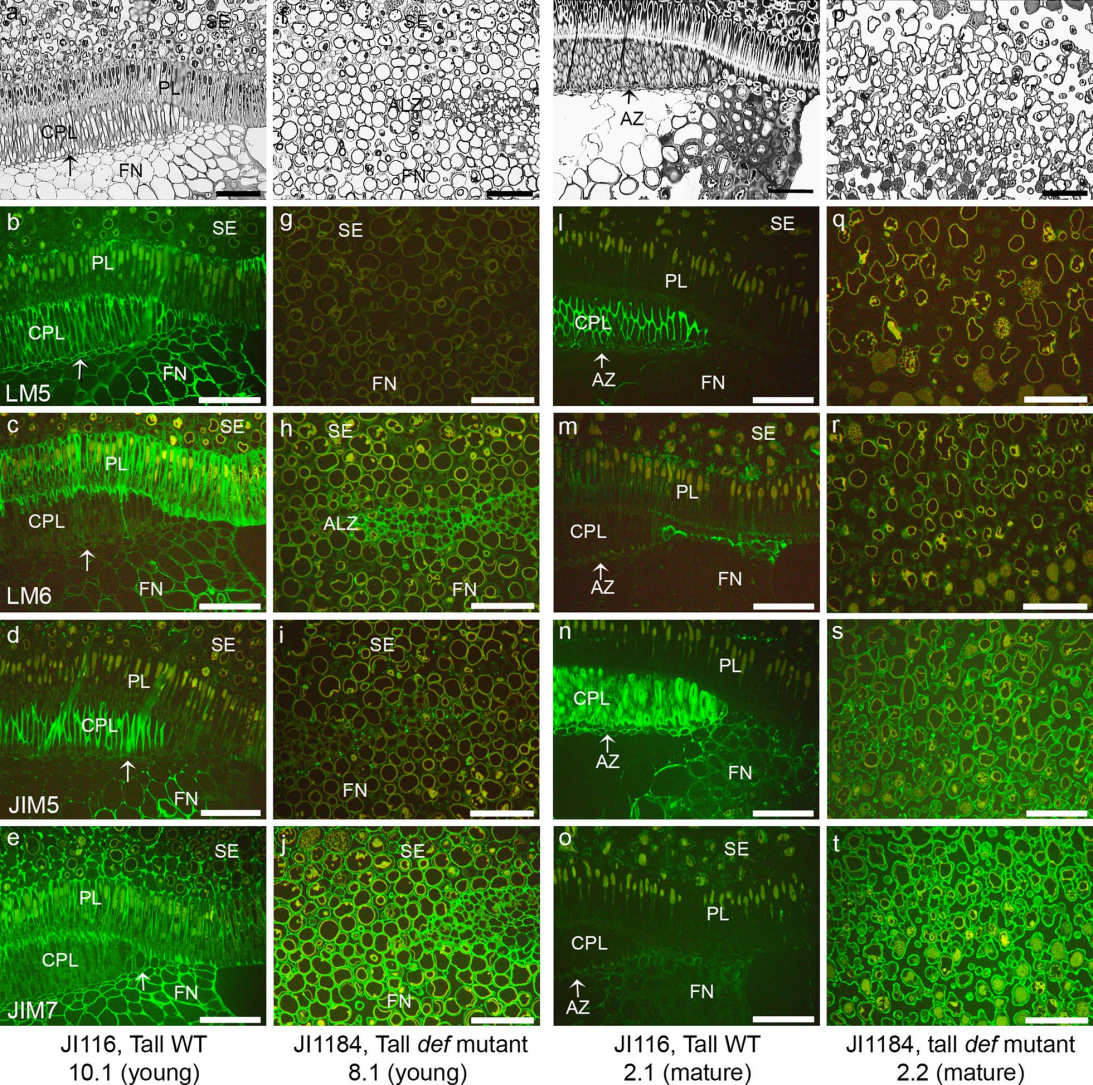
Light micrographs of sections stained with *toluidine blue* to show structural differences (**a**, **f**, **k** and **p**) and micrographs of indirect immunofluorescence detection of pectic epitopes in AZ of tall wild type (JI116) and in ALZ of tall *def* mutant (JI1184) pea seeds. **a**–**e** Tall wild type young seeds (JI116 10.1). **f**–**j** Tall *def* mutant young seed (JI1184 8.1). **k**–**o** Tall wild type mature seed (JI116 2.1). **p**–**t** Tall *def* mutant mature seed (JI1184 2.2). **b**, **g**, **l** and **q** Immunolabeling with LM5 galactan. **c**, **h**, **m** and **r** Immunolabeling with LM6 arabinan. **d**, **i**, **n** and **s** Immunolabeling with JIM5. **e**, **j**, **o** and **t** Immunolabeling with JIM7. *PL* Palisade layer, *CPL* Counter palisade layer, *AZ* abscission zone; *FN* funicle; *SE* seed; *ALZ* abscission less zone. *Arrows* indicated the AZ. *Scale bars* 100 μm

**Fig. 3:**
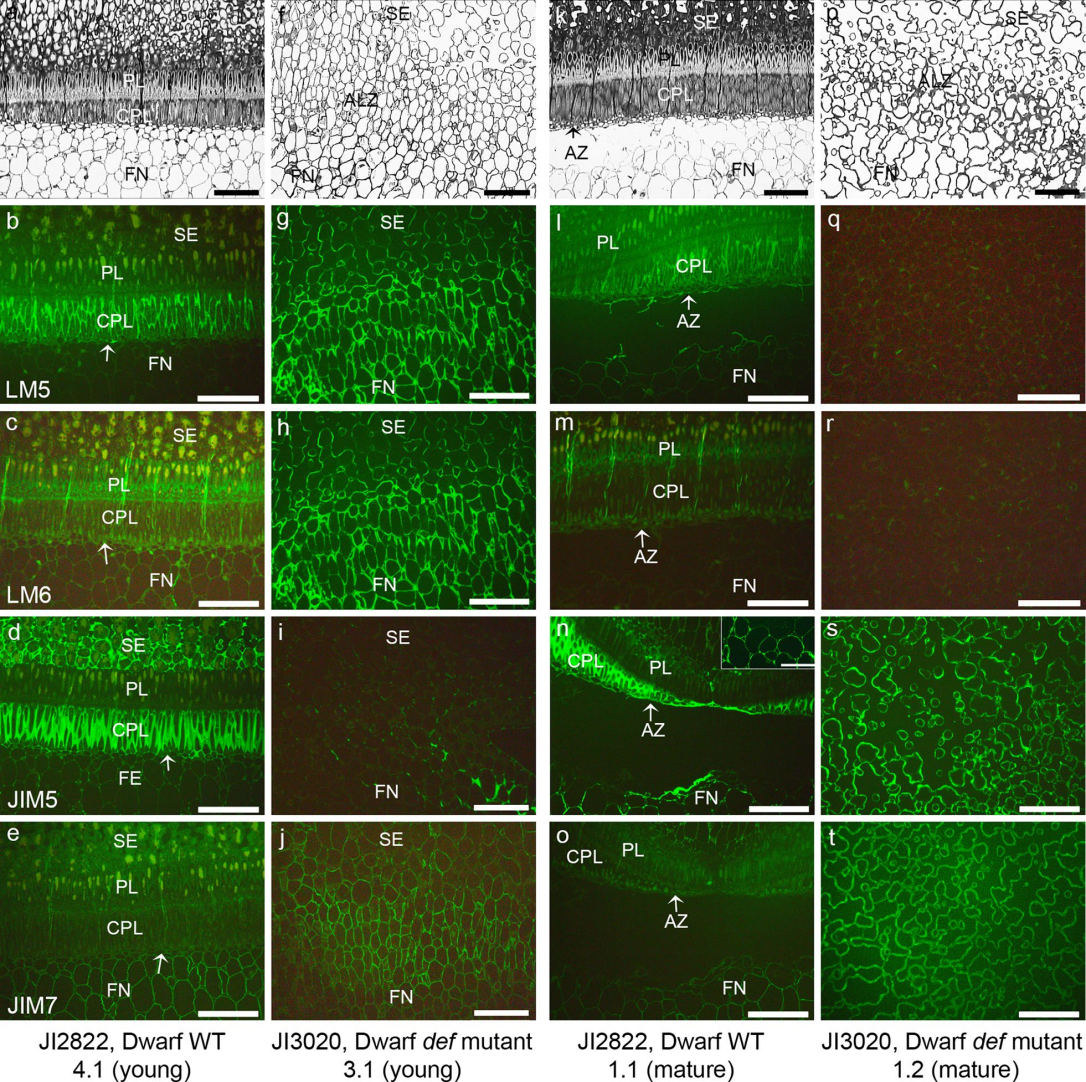
Light micrographs of sections stained with *toluidine blue* to show structural differences (**a**, **f**, **k** and **p**) and micrographs of indirect immunofluorescence detection of pectic epitopes in the AZ of dwarf wild type (JI2822) and ALZ of dwarf *def* mutant (JI3020) pea seeds. **a**–**e** Dwarf wild type young seeds (JI2822). **f**–**j** Dwarf *def* mutant young seed (JI3020). **k**–**o** Dwarf wild type mature seed (JI2822). **p**–**t** Dwarf *def* mutant mature seed (JI3020). **b**, **g**, **l** and **q** Immunolabeling with LM5 galactan. **c**, **h**, **m** and **r** Immunolabeling with LM6 arabinan. **d**, **i**, **n** and **s** Immunolabeling with JIM5. **e**, **j**, **o** and **t** Immunolabeling with JIM7. *PL* palisade layer; *CPL* counter palisade layer; *AZ* abscission zone; *FN* funicle; *SE* seed; *ALZ* abscission less zone. *Arrows* indicated the AZ. *Scale bars* 100 μm

To study the spatial and developmental distribution of the pectic polysaccharides during the abscission process in the tall WT pea and non-abscission tall *def* mutant, indirect immunolabeling was performed using (1 → 4)-β-d-galactan (LM5), (1 → 5)-α-l-arabinan (LM6), partially de-methyl esterified HG (JIM5) and methyl esterified HG (JIM7) antibodies (Fig. 2). To study developmental difference in the abscission event, we used young seeds that did not develop the AZ yet (Fig. 2a–e) of the tall WT pea JI116 seed and the mature pea seeds that developed entire abscission event (Fig. 2k–o). The young pea seed did not show any AZ (Fig. 2a). The LM5 epitopes mostly localized in every area in the young seeds including funicles and funicle-seed coat interface (Fig. 2b). The LM6 epitopes were found in the palisade layer (PL) and cell walls in the funicle of the young seeds (Fig. 2c). The young seed showed partially de-methyl esterified JIM5 epitopes in the CPL and part of the funicles but the interface between seed coat and the funicle did not show JIM5 epitopes (Fig. 2d). Usually JIM7 epitope was found over whole tissues (Fig. 2e) in the young WT pea seed. To compare the cell wall components between the WT pea and the *def* mutant pea seeds, we used JI1184 for the tall mutant. The *def* mutant pea seed showed irregular cells in the abscission less zone (ALZ) and there was no distinct borderline between the pea seeds and the funicles (Fig. 2f and p). Usually the LM5 epitopes were not seen in the young pea seeds (Fig. 2g) while the LM6 epitopes were abundant in the young *def* mutant pea seeds (Fig. 2h). The young mutant pea seed showed less labeling of the JIM5 (Fig. 2i). The JIM7 epitopes were evenly detected in the young mutant pea seeds (Fig. 2j). The mature pea seed showed distinct AZ development in the wild type (Fig. 2k) but there was abscission less zone in the mutant (Fig. 2p). The mature seeds of the WT showed the LM5 immunolabeling only in the CPL and no LM5 epitopes in the AZ cells (Fig. 2i). In the mature WT pea seeds, the LM6 epitopes disappeared in the PL and the cell walls in the AZ (Fig. 2m). In the mature WT pea seeds, the cells in the AZ showed strong JIM5 labeling, especially the cells in the CPL, interface between seed coat and funicle and few cell layers in the funicels closely related to the AZ (Fig. 2n). The mature seed showed very weak labeling of the JIM7 (Fig. 2o). The mature *def* mutant pea did not show neither LM5 nor LM6 epitopes (Fig. 2q and r). However, the mature *def* mutant pea seed had abundant JIM5 epitope in the ALZ (Fig. 2s). The JIM7 epitopes were evenly detected both in the mature *def* mutant pea seeds (Fig. 2t). To get the F_3_ populations, we crossed the dwarf WT (JI2822) and the mutant types (both tall and dwarf). Therefore, it is necessary to identify that the dwarf WT revealed similar labeling pattern as in the tall WT (JI116). The dwarf WT pea (JI2822) showed mostly similar feature with the tall WT pea (JI116) (Fig. 3). The dwarf WT pea seed (JI2822) had the distinct AZ in the mature and young seeds (Fig. 3k and c, although Fig. 3a did not show the AZ in this figure). The young seed (JI2822 4.1) showed the LM5 epitope in the CPL and a bit in the PL. However, the cells in the interface between the seed coat and the funicles did not show any LM5 epitope, although this area did not have the AZ (Fig. 3b). The LM6 epitopes were detected in the PL, partially in the CLP and cells in the funicle in the young pea seeds (Fig. 3c). The JIM5 epitopes were easily detected in the CPL of the young seeds (Fig. 3d). The JIM7 epitopes were distributed in the young pea seeds (Fig. 3e). The *def* dwarf mutant JI3020 pea seeds showed irregular cells in the ALZ and there was no distinct borderline between the pea seeds and the funicles as shown in the tall mutant (Fig. 3f and p). Usually the LM5 and the LM6 epitopes were seen in the young *def* mutant pea (Fig. 3g and h). The young mutant pea seed showed less labeling of the JIM5 (Fig. 3i). However, the JIM7 epitope was detected in the young mutant pea seeds (Fig. 3j). The mature seeds (JI2822 1.1) revealed the reduced LM5 epitope in the CPL and the cells in the interface between the seed coat and the funicle (Fig. 3l). The LM6 epitopes were not detected in the CPL and funicle of the mature seeds (Fig. 3m). The cells in the funicle especially near to the AZ and CPL in the mature WT pea seeds were strongly immunolabeled with the JIM5 (Fig. 3n and insert). The mature WT pea seed, especially the cells in the CPL and the FN did not show the JIM7 epitopes (Fig. 3o). The LM5 and LM6 epitopes were not detected in the ALZ in the mature *def* mutant (Fig. 3q and r). However, the mature *def* mutant pea seed showed the abundant JIM5 epitopes in the ALZ (Fig. 3s) and the JIM7 epitopes were also easily detected in the mature mutant pea seeds (Fig. 3t).

**Fig. 4:**
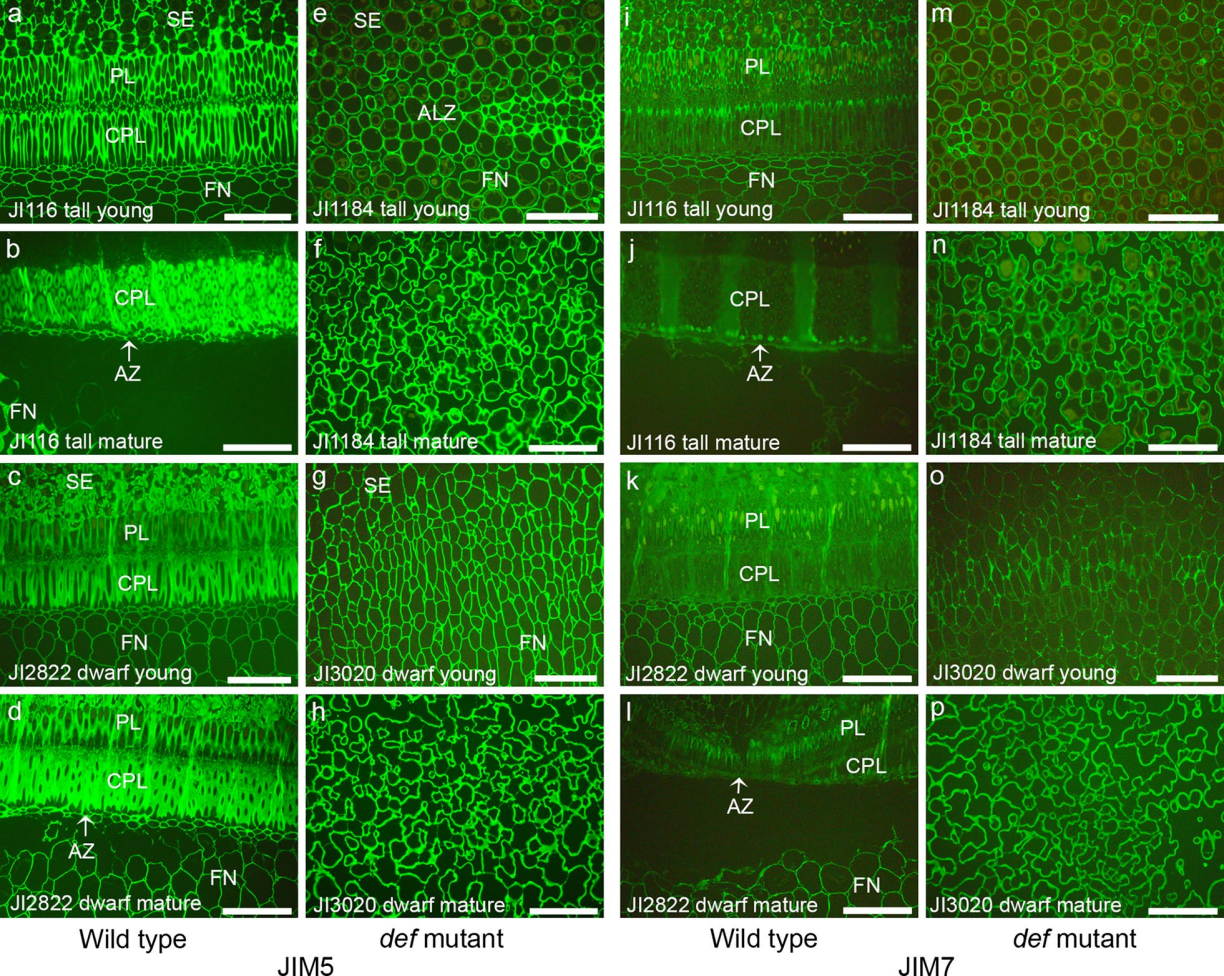
Micrographs of indirect immunofluorescence detection of pectic epitopes JIM5 and JIM7 in wild type (JI116 and JI2822) and *def* mutant (JI1184 and JI3020) pea seeds. Prior to labeling, longitudinal sections were treated with 0.05 M Na_2_CO_3_ for de-esterification of homogalacturonan. **a**–**h** Immunolabeling with JIM5. **i**–**p** Immunolabeling with JIM7. **a** and **i** Tall wild type young pea seeds (JI116 at 10.1). **b** and **j** Tall wild type mature pea seeds (JI116 at 2.1). **c** and **k** Dwarf wild type young pea seeds (JI2822 at 4.1). **d** and **i** Dwarf wild type mature pea seeds (JI2822 at 1.1). **e** and **m** Tall *def* mutant young pea seeds (JI1184 at 8.1). **f** and **n** Tall *def* mutant mature pea seeds (JI1184 at 2.2). **g** and **o** Dwarf *def* mutant young pea seeds (JI3020 3.1).**h** and **p** Dwarf *def* mutant mature pea seeds (JI3020 1.2). *PL* palisade layer; *CPL* counter palisade layer; *AZ* abscission zone; *FN* funicle, *SE* seed; *ALZ* abscission less zone. *Arrows* indicated the AZ. *Scale bars* 100 μm

To see the degree of de-esterification, sections were treated with Na_2_CO_3_ prior to immunolabeling (Fig. 4). After de-esterification, all of the materials were strongly labeled with the JIM5 antibody. Abundant JIM5 epitopes in the PL of the young seeds after Na_2_CO_3_ treatment indicate highly methyl esterified HG in this area in nature (Fig. 4a and c). Strongly de-esterified young mutant seed cell walls after Na_2_CO_3_ treatment (Fig. 4e and 4g) suggest that the young mutant seed cell walls are highly methyl esterified in nature. The JIM7 epitopes were rarely detected in the mature WT (Fig. 4j and l) but were present in the young WT pea seeds (Fig. 4i and k). All the mutant pea seeds showed even distribution of the JIM7 epitopes (Fig. 4m–p). Together, these results indicate that the young mutant pea seeds are highly methyl esterified and the mature mutant pea seeds are partially de-esterified in nature.

### Pectic polysaccharide distribution in F_3_ population from a cross of the dwarf WT JI 2822 and the tall *def* mutant JI 1184

**Fig. 5:**
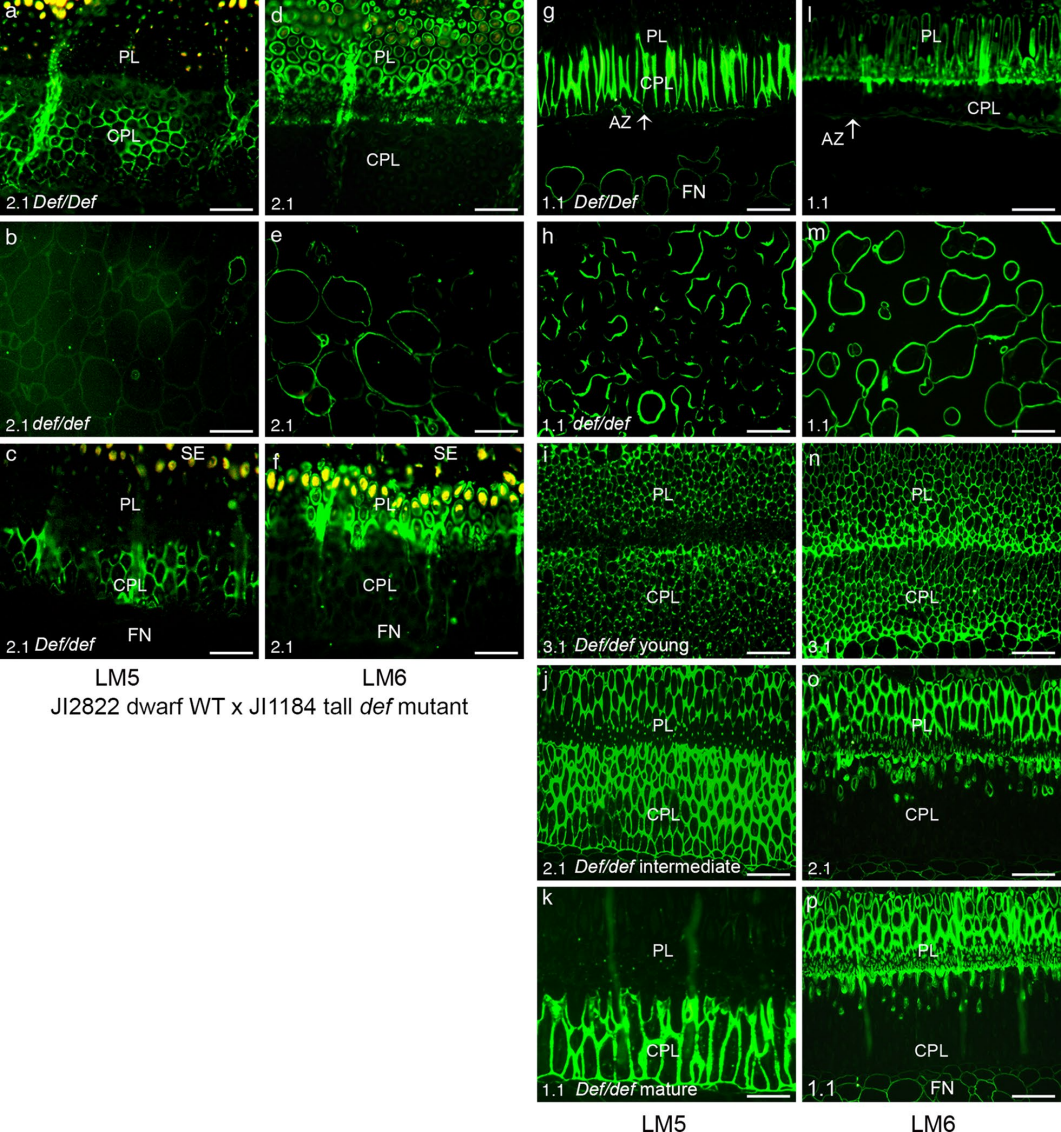
Micrographs of indirect immunofluorescence detection of pectic epitopes LM5 galactan and LM6 arabinan in F_3_ population from cross of dwarf wild type JI 2822 and tall *def* mutant JI 1184 at pod number 2 stage (**a**–**f**) and F_3_ population from cross of the dwarf wild type JI 2822 and dwarf *def* mutant JI 3020 (**g**–**p**). **a**–**c** and **g**–**k** Immunolabelling with LM5 galactan. **d**–**f** and **l**–**p** Immunolabelling with LM6 arabinan. **a** and **d** Homozygous dominant (*Def/Def*) F_3_ line 11 showing the presence of double palisade layers as in the wild type phenotype. **b** and **e** Homozygous recessive (*def/def*) F_3_ line 18 showing the presence of the ALZ as in *def* mutant phenotypes. **c** and **f** Heterozygous (*Def/def*) F_3_ line 14 showing the presence of PL and CPL. **g** and **l** Homozygous dominant (*Def/Def*) F_3_ line 1 showing the presence both PL and CPL as in the WT phenotypes. **h** and **m** Homozygous recessive (*def/def*) F_3_ line 33 showing the ALZ as in parents *def* mutant phenotypes. **i** and **p** Heterozygous (*Def/def*) F_3_ line 77. **i** and **n** Young seed at pod stage 3.1. **j** and **o** Intermediated seed at pod stage 2.1. **k** and **p** Mature seed at pod stage 1.1. *PL* palisade layer; *CPL* counter palisade layer; *AZ* abscission zone; *FN* funicle; *SE* seed. *Scale bars* 50 μm

To study the mapping of pectic epitopes in F_3_ populations, a homozygous dominant line 11 (*Def*/*Def*), a homozygous recessive line 18 (*def*/*def*) and a heterozygous line 14 (*Def/def*) were tested. The homozygous dominant line 11 (*Def*/*Def*) showed a well-defined double palisade layers and a clear abscission process while the homozygous recessive line 18 (*def*/*def*) had no double PLs and no abscission. However, the heterozygous line 14 (*Def/def*) exhibited partially developed double PLs [41]. The objective of the indirect immunolabeling using the LM5 and the LM6 antibodies was to visualise the distribution of these pectic polysaccharides in different cross lines upon the segregation pattern of the *Def* locus, involved in the seed abscission from the funicle. The homozygous dominant line 11 showed the distinct LM5 labeling in the CPL as seen in parents phenotypes (Fig. 5a) while the homozygous recessive line 18 (*def*/*def*) did not show any labeling of LM5 in the ALZ (Fig. 5b). However, the heterozygous line 14 (*Def/def*) showed partial LM5 labeling in the CPL (Fig. 5c). When LM6 antibody was used for the indirect immunolabeling in the F_3_ lines, the palisade layer in the homozygous dominant line 11(*Def*/*Def*) was labeled with the LM6 antibody in the PL but not in the CPL (Fig. 5d). There was labeling of the LM6 in the cell walls comprising the ALZ in the homozygous recessive line 18 (*def*/*def*) (Fig. 5e). The CPL in the F_3_ heterozygous line 14 (*Def/def*) was not labeled with the LM6, but the PL was partially labeled (Fig. 5f). Thus, we identified that the patterns of the pectic polysaccharide distribution in the F_3_ population were similar to its parents.

### Pectic polysaccharide distribution in F_3_ population from a cross of the dwarf WT JI 2822 and the dwarf *def* mutant JI 3020

We further examined the immunolabeling patterns using the LM5 and the LM6 antibodies in the second F_3_ population from the cross of the dwarf WT JI 2822 and the dwarf *def* mutant JI 3020 to confirm segregation of the *Def* locus involved in the seed abscission. The LM5 epitopes were observed in the CPL in the homozygous dominant line 1 (*Def/Def*) and this labeling pattern was similar to the parents dwarf WT JI 2822 (Fig. 5g). The homozygous recessive line 33 (*def*/*def*) showed neither an abscission process nor well-defined PLs. The homozygous recessive line showed the ALZ and intense labeling of the LM5 was detected in the ALZ (Fig. 5h). In contrast, the F_3_ heterozygous line 77 (*Def/def*) showed an interesting differentiation of the PLs and the labeling pattern. In the young seed at the development stage 3.1, we observed the LM5 labeling in the both PLs, although the PL was not distinctively differentiated (Fig. 5i). At the intermediate stage of the line 77 (stage 2.1), the LM5 labeling began to disappear in the PL while the CPL was still intensely labeled and the differentiation of the palisade layers were more distinct (Fig. 5j). At the mature developmental stage 1.1, the LM5 epitopes were detected only in the CPL and not in the PL as shown in the homozygous dominant line 1 (*Def/Def*) (Fig. 5k). When the anti-arabinan antibody LM6 was used in the second F_3_ population, only the PL was labelled in the homozygous dominant line 1 (*Def/Def*) (Fig. 5l). In the homozygous recessive line 33 (*def*/*def*), the ALZ was labeled with the LM6 (Fig. 5m). Interestingly, the heterozygous line 77 (*Def/def*) at the young developmental stage 3.1 revealed a distinct labeling of the LM6 arabinan epitope in the both PL and CPL (Fig. 5n). However, the intermediate developmental stage 2.1 showed partial labeling of the LM6 in the CPL (Fig. 5o) and the mature stage (1.1) showed very less labeling of the LM6 epitopes in the CPL (Fig. 5p). Together with the LM5 labeling, we suggest that the LM5 and LM6 labeling are highly dependent on seed maturity and futher abscission process.

## Discussion

In our previous work, the structural analysis of the WT showed the clear abscission event that was associated with the distinct double palisade layers at the junction between the seed coat and the funicle [4]. However, the *def* mutant pea seeds failed to abscise from the funicle and the double palisade layers were completely absent. Therefore, we conclude that the presence of the double palisade layers in the WT pea seeds plays an important structural role in the AZ formation. Furthermore, the F_3_ populations also showed typical structural differentiation in the both crossings [41]. Usually, structural changes during abscission process accompany with cell wall modification as we previously studied in poinsettia [3]. Unlike poinsettia, the pea seeds have predestined primary AZ between the seed coat and the funicles [42]. Does the predestined AZ in the WT pea seeds undergo similar way in cell wall modification as in the secondary abscission in poinsettia? Moreover, does the *def* mutant pea seeds have different cell wall components leading the non-abscission? To answer these questions, we investigated the temporal and spatial distribution of the pectic epitopes in cell walls of the WT pea seeds and *def* mutant pea seeds. We further examined immunolabeling patterns in the F_3_ populations resulting from the crosses between the WT and the mutant parents.

The LM5 galactan was found in the WT young seeds in the PL, the CPL and cells in the funicles (Fig. 2b). However, the LM5 epitopes were reduced in the seed and the funicles even in the CPL during the abscission process (Fig. 3b) although those seeds did not show the abscission. This is an interesting indication. In poinsettia flower pedicel, the LM5 galactan disappeared dramatically in early stage of the abscission process although the AZ did not occur. Thus, reduction of galactan in the WT pea seeds may be an important sign for the abscission. The LM6 arabinan epitopes were also reduced in the mature pea seeds during the abscission. Usually, the LM6 epitopes are localized in the PL and the funicle, but as the abscission proceeded the LM6 epitopes disappeared in these areas. A significant reduction of LM5 and LM6 epitopes in the cell walls of the mature seeds suggests that the loss of the galactan and the arabinan in the cell walls reflects remodeling of the cell wall during the abscission and that the cell wall compositional changes occur prior to the visibly recognizable abscission [3]. Both the PL and the CPL have been shown to strengthen the attachment of the seed to the funicle [18]. The loss of the LM5 galactan and the LM6 arabinan epitopes during the abscission process may suggest a clue for double palisade layers in their structural role in the abscission. The CPL is the region located exactly above the AZ and the loss of the LM5 and the LM6 epitopes in this area may give a chance to modify the cell walls that induce easy break down of the cell walls resulting in cell separation, like in the poinsettia flower abscission [3]. The differential occurrence of the galactan and the arabinan in the PL and the CPL respectively suggests a spatial regulation of the RG-I side chains as reported for tomato fruit pericarp [43].

The reduction or loss of the LM5 and the LM6 epitopes may possibly result from the enzyme actions. Many investigations support the idea that the putative hydrolytic enzyme(s) such as galactanase and arabinase may be involved in the loss of cell wall components and weakening the cell wall networks during abscission [44–46]. Furthermore, the degradation of the RG-I backbone makes naked HG backbone that can be easily affected by pectin methyl esterase (PME), PME inhibitor or polygalacturonase (PG) that may directly or indirectly function on the cell wall network modification [10, 44, 47]. The PME functions to remove the methyl group from the HG backbone and make the HG easy to be degraded by the polygalacturonase [48]. However, the de-methyl esterification of the HG is not only considered in the cell wall weakness. The de-esterified HG is readily cross linked by calcium resulting in stiffer walls and modify the mechanical properties of the cell walls [30, 49]. Thus, the de-methyl esterifican promotes cell adhesion in one hands and cell separation in another hands. Abundant of the JIM5 epitopes in the AZ indicated that increased cell wall rigidity to protect tissues after organ separation. In the poinsettia flower abscission, the JIM5 HG epitopes were abundant in the AZ and least abundant in the proximal area that is attached to the mother plants [3]. Interesting dynamic of the JIM5 epitopes were observed in the *def* mutant pea seeds. Usually the mature mutant pea seeds showed more de-esterified HG than the WT pea seeds or the young *def* mutant pea seeds. The de-esterification by Na_2_CO_3_ treatment showed that the young mutant pea seeds cell walls were highly methyl esterified HG. The de-methyl esterification of the HG in the mature *def* mutant seeds, can potentially contribute to the rigidity of the wall structure. Thus, the reduced methyl esterification in the HG backbone increases the potential for cross-links and leads to a more rigid gel with increased visco-elastic properties and hardness [30, 50–52]. Therefore, we suggest that the structural assembly such as absence of the double palisade layers and biochemical compositional differences of the cell walls such as de-methyl esterification may lead non-abscission in the *def* mutant pea seeds.

As we expected, there was not a remarkable change in the *def* mutants in their cell wall components upon seed maturity. However, the cross of the WT and the *def* mutant showed interesting aspects. The homozygous dominant line 11 (*Def*/*Def)* showed similar structure and similar labeling pattern to their WT parents. The homozygous recessive line 18 (*def*/*def*) showed also similar structural and immunolabeling pattern in the ALZ of the tall *def* mutant. The heterozygous line 14 (*Def*/*def*) showed partial AZ formation and a similar labeling pattern of the LM5 and the LM6 to the parents. The immunolabeling analysis suggests a monogenic pattern of inheritance of the *Def* locus in the WT involved in seed abscission whilst the homozygous recessive (*def*/*def*) is characterized by the non-abscission. The second F_3_ population from the cross dwarf WT JI 2822 and dwarf *def* mutant JI 3020 revealed more interesting findings. The homozygous dominant line 1 (*Def*/*Def*) showed similar pattern to the WT parents but the homozygous recessive line 33 (*def/def*) showed the non-abscission. However, in the heterozygous line 77 (*Def/def*), the structure and the immunolabeling pattern showed differences upon seed maturity. Through these results, we assume that the galactan and the arabinan localization is regulated by the seed maturity and futher abscission. McCartney et al. [32] reported that the distribution of the LM5 and the LM6 epitopes is related to the plant development. In several plants, senescence accompanies the abscission process [53, 54]. Therefore, it is not surprising that the compositional changes in the galactan and the arabinan are dependent on the seed maturity and the abscission.

## Conclusions

Our results contribute new insights into understanding the structural and architectural organization of the abscission processes in the WT pea seeds and the ALZ in the *def* mutant pea seeds through the study of the complexity and variability of pectins in plant cell walls as well as understanding the segregation patterns of the *Def* locus through immunolabeling studies. We see clear similarities between the earlier described secondary (induced) abscission process in poinsettia [3] and the primary abscission (preformed) we see here in pea. This indicates universal cell wall alterations in abscission, regardless of whether it is the primary or the secondary, with poinsettia and pea, being separated by 94–98 million years of evolution [55].

## Abbreviations

WT: wild type
HG: homogalacturonan
AZ: abscission zone
*Def*: development funiculus
PL: palisade layer
CPL: counter palisade layer
XGA: xylogalacturonan
RG-I: rhamnogalacturonan-I
RG-II: rhamnogalacturonan-II
Gal A: galacturonic acid
mAbs: monoclonal antibodies
ALZ: abscission less zone
PBS: phosphate-buffered saline
PME: pectin methyl esterase
PG: polygalacturonase
